# Skeletal muscle anabolism is a side effect of therapy with the MEK inhibitor: selumetinib in patients with cholangiocarcinoma

**DOI:** 10.1038/bjc.2012.144

**Published:** 2012-04-17

**Authors:** C M M Prado, T Bekaii-Saab, L A Doyle, S Shrestha, S Ghosh, V E Baracos, M B Sawyer

**Affiliations:** 1 Department of Oncology, University of Alberta, Cross Cancer Institute, 11560 University Avenue, Edmonton, Alberta T6G 1Z2, Canada; 2 Department of Internal Medicine, The Ohio State University Comprehensive Cancer Center, A454 Starling Loving Hall, 320 West 10th Avenue, Columbus, OH 43210, USA; 3 Department of Pharmacology, The Ohio State University Comprehensive Cancer Center, A454 Starling Loving Hall, 320 West 10th Avenue, Columbus, OH 43210, USA; 4 Division of Cancer Treatment and Diagnosis, NCI, Executive Plaza North, Suite 7122 6130 Executive Blvd., Bethesda, MD 20892-7426, USA

**Keywords:** cholangiocarcinoma, skeletal muscle, cachexia, interleukin-6

## Abstract

**Background:**

Cancer cachexia is characterised by skeletal muscle wasting; however, potential for muscle anabolism in patients with advanced cancer is unproven.

**Methods:**

Quantitative analysis of computed tomography images for loss/gain of muscle in cholangiocarcinoma patients receiving selumetinib (AZD6244; ARRY-142886) in a Phase II study, compared with a separate standard therapy group. Selumetinib is an inhibitor of mitogen-activated protein/extracellular signal–regulated kinase and of interleukin-6 secretion, a putative mediator of muscle wasting.

**Results:**

Overall, 84.2% of patients gained muscle after initiating selumetinib; mean overall gain of total lumbar muscle cross-sectional area was 13.6 cm^2^/100 days (∼2.3 kg on a whole-body basis). Cholangiocarcinoma patients who began standard treatment were markedly catabolic, with overall muscle loss of −7.3 cm^2^/100 days (∼1.2 kg) and by contrast only 16.7% of these patients gained muscle.

**Conclusion:**

Our findings suggest that selumetinib promotes muscle gain in patients with cholangiocarcinoma. Specific mechanisms and relevance for cachexia therapy remain to be investigated.

Cholangiocarcinoma is an uncommon cancer that is associated with a dismal prognosis and significant weight loss and muscle wasting (cancer cachexia; Cooperman *et al*, 2000; Mosconi *et al*, 2009). A hallmark of this disease is elevated serum interleukin-6 (IL-6; Goydos *et al*, 1998) levels, a proinflammatory cytokine that also elicits protein catabolism in skeletal muscle (Bruce and Dyck, 2004). Muscle wasting is a defining feature of cancer cachexia and has major impacts on physical and respiratory function, immunity, chemotherapy response and overall survival (MacDonald *et al*, 2003; Prado *et al*, 2008, 2009; Saini *et al*, 2009; Dodson *et al*, 2011). Owing to the importance of muscle mass in physiological function and association between muscle loss and outcomes of cancer, alterations in muscle mass as a side effect of anticancer agents is of growing interest. Intracellular signals involved in skeletal muscle anabolism and catabolism have been elucidated. PI3K, AKT and mTOR are central to activating muscle protein synthesis by amino acids (Bodine *et al*, 2001; Edinger and Thompson, 2002; Saini *et al*, 2006; Durham *et al*, 2009). Induction of muscle anabolism by physical activity occurs by pathways involving RAF, MEK and MAPK/ERK kinases (Bodine *et al*, 2001; Fearon *et al*, 2011). Cancer therapies directed at these targets would be expected to provoke muscle wasting and this was shown for sorafenib (Antoun *et al*, 2010). By contrast, some mitogen-activated protein/extracellular signal–regulated kinase kinase (MEK) inhibitors in the development for cancer therapy are anti-inflammatory. Selumetinib (AZD6244, ARRY-142886; AstraZeneca, Manchester, UK), an allosteric inhibitor of MEK1 and MEK2 phosphorylation of ERK (Bekaii-Saab *et al*, 2011), has tumour suppressive activity in preclinical models (Revill *et al*, 2006) and has been proven to inhibit IL-6 production (Tai *et al*, 2007). As proinflammatory cytokines promote muscle protein catabolism (Zaki *et al*, 2004; Argiles *et al*, 2009; Murphy and Lynch, 2009), and IL-6 is considered one of the principal catabolic actors in skeletal muscle (Bruce and Dyck, 2004), such agents may mitigate muscle wasting.

In our recent phase II trial of selumetinib (Bekaii-Saab *et al*, 2011), patients receiving selumetinib experienced an average of 3.9 kg confirmed nonfluid weight gain. Considering the observed weight gain of patients in our phase II study, we investigated muscle and/or fat tissue gain using computed tomography (CT) as described below. The comparator group included patients with advanced cholangiocarcinoma who received standard therapies.

## Materials and methods

Studies were approved by Research Ethics Boards of Ohio State University and Alberta Cancer Board.

### Selumetinib treatment group

Patients with advanced cholangiocarcinoma participated in a phase II study of selumetinib (100 mg PO b.i.d.; Bekaii-Saab *et al*, 2011). The formulation was selumetinib-free base in a liquid vehicle Captisol (sulpha-butyl-ethyl B-cyclodextrin). Study inclusion and exclusion criteria have previously been published (Bekaii-Saab *et al*, 2011).

### Standard therapy group

The Cross Cancer Institute is the only cancer centre serving northern Alberta, Canada (population: 1 800 000). A database of all cases (Alberta Cancer Registry) codes primary cancers by site, morphology, clinical and demographic information. For this study, all invasive cholangiocarcinoma cases diagnosed between 1997 and 2007 and included in the Cancer Registry were identified (ICD-10 MO codes: 8140/3, 8141/3, 8160/3, 8162/3, 8180/3) and these were included if they had been evaluated by CT at diagnosis and at least once after starting treatment.

No patients in either group were prescribed anabolic interventions for anorexia-cachexia syndrome (e.g., megesterol acetate, oxandrolone or corticosteroids).

### Body composition measurements

Digitally stored CT scans were analysed using Slice-O-Matic software V4.2 (Tomovision, Montreal, Canada). The directly determined measure was cm^2^ of total skeletal muscle and total adipose tissue at the third lumbar vertebra (L3), a bony landmark previously validated (Mourtzakis *et al*, 2008) and utilised (Prado *et al*, 2007, 2008, 2009) in studies of cancer patients. The precision error of measurements is ∼1.5% (Mourtzakis *et al*, 2008) with a minimum detectable change of approximately 3 cm^2^.

Changes in muscle or adipose tissue are reported as mean cm^2^ (s.d.) lost or gained over time and also divided into three categories: (A) loss ⩾6.0 cm^2^, (B) stable ±5.9 cm^2^ or (C) gain ⩾6.0 cm^2^ of muscle. These cutoffs are equivalent to loss/gain of ⩾1 kg of skeletal muscle on a whole-body basis (Shen *et al*, 2004), which are of sufficient magnitude to associate with alterations in muscle strength (Frontera *et al*, 1988). For adipose tissue, categories were based on the equivalence of 14.7 cm^2^ total fat at L3 and 1 kg tissue on a whole-body basis (Shen *et al*, 2004).

### Statistics

Data are expressed as mean±s.d. or median/s.e. for continuous variables. Comparisons for categorical variables were conducted using test of proportions, while Student’s *t*-test was used for continuous variables. Kaplan–Meier curves and log-rank tests were used to compare study groups in relation to survival. Analysis was conducted using SPSS software version 18.0 (SPSS, Chicago, IL, USA). All *P*-values were two-sided and levels of significance were *P*<0.05.

## Results

Demographics of study participants are described in Table 1. A total of 20 patients from the selumetinib phase II study had images that included the third lumbar vertebra. Patients with cholangiocarcinoma receiving standard treatment (*n*=30) received the following treatments for either first- or second-line therapy: carboplatin, paclitaxel, etoposide (*n*=4), gemcitabine with or without capecitabine (*n*=6), epirubicin, carboplatin, capecitabine (*n*=4), and radiation (*n*=7). Nine patients received best supportive care.

The mean interval between scans was 91.5 days for selumetinib-treated patients and 85.5 days for cholangiocarcinoma patients. To account for variation in the exact duration of scan intervals, changes in tissue areas are expressed as: (cm^2^ lost or gained/number of days between scans) × 100.

**Figure 1:**
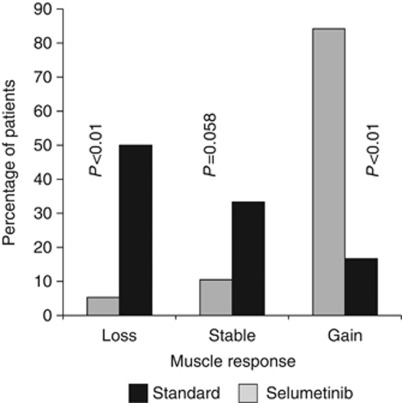
Percentage of patients exhibiting loss >1 kg, no change and gain >1 kg of skeletal muscle after initiation of selumetinib therapy or standard treatment. *P*-value calculated using test of proportions.

Overall, selumetinib-treated cholangiocarcinoma patients gained skeletal muscle, in contrast to those receiving standard therapy, who were markedly catabolic (Table 1, Figure 1); 84.2% of patients gained muscle after initiating selumetinib, compared with 16.7% of patients who were on standard treatment (*P*<0.001, Figure 1). Selumetinib-treated patients muscle cross-sectional area increased by +13.8 (11.9)cm^2^/100 days compared with a loss of −7.3 (14.3) cm^2^/100 days for non-selumetinib-treated patients (*P*<0.001; Table 1). This translates to approximately +2.3 *vs* −1.2 kg of skeletal muscle on a whole-body basis, respectively. Tissue gains noted for selumetinib-treated patients were restricted to skeletal muscle (Table 1). Adipose tissue was lost in both groups. There were no observed differences in muscle or adipose tissue changes (gain, stable or loss) between men and women in the standard therapy group *vs* the selumetinib group (*P*=0.478 for muscle change and *P*=0.557 for adipose tissue change).

**Figure 2:**
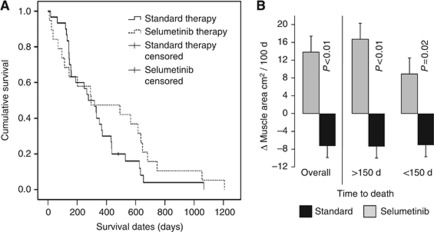
Comparison of survival and muscle response to selumetinib therapy or standard treatment in relation to time to death. (**A**) Survival of patients on selumetinib therapy or on standard treatment. *P*-value calculated by log-rank test. (**B**) Mean loss or gain of total lumbar skeletal muscle (cm^2^) during treatment, overall and stratified by time to death. *P*-value calculated by *T*-test.

Survival of the two groups is illustrated in Figure 2a. Median time to death was not different between selumetinib *vs* standard therapy (Table 1, Figure 2A). Because the likelihood of muscle loss increases as death approaches (Lieffers *et al*, 2009), the selumetinib and standard therapy patients were further compared after stratification by time to death (Figure 2B). Regardless whether patients were started on selumetinib within 150 days of death or earlier, the selumetinib-treated patients showed significant gain of skeletal muscle compared with the standard care group.

## Discussion

Cholangiocarcinoma is one of the most lethal cancers and is typically associated with cachexia. We show that selumetinib, an agent that holds promising activity in cholangiocarcinoma (Bekaii-Saab *et al*, 2011), induces rapid and significant skeletal muscle gain. Muscle gain is unanticipated in advanced biliary cancer and was not observed in our comparator group of cholangiocarcinoma patients on standard therapy.

Selumetinib may have direct or indirect action on muscle. Muscle contains both MEK 1 and 2, which are involved in the promotion of myogenic differentiation (Jo *et al*, 2011). Selumetinib has also been shown to inhibit secretion of cytokines such as IL-6 (Tai *et al*, 2007), IL-1*β* and tumour necrosis factor-*α*, which are implicated in the promotion of cancer cachexia (Zhang *et al*, 2007, 2008). While the mechanism of action for this anabolic reaction for selumetinib remains unproven, it seems likely that the observed increase in muscle is related to inhibition of cytokine secretion, as inhibition of MEK1/2 would be expected to actually inhibit muscle growth. We previously showed that another tyrosine kinase inhibitor, sorafenib, for example, provokes muscle loss in a randomised, placebo-controlled study (Antoun *et al*, 2010). In contrast, our current results indicate that the weight gain associated with selumetinib treatment (Bekaii-Saab *et al*, 2011) is related to increased muscle mass.

Neither patients treated with selumetinib nor those treated standard care group gained adipose tissue. This is consistent with published data demonstrating that muscle and fat are not necessarily gained or lost in concert (Prado *et al*, 2008). Additionally, a recent international consensus definition of cancer cachexia has characterised cachexia by muscle loss occurring with or without the loss of adipose tissue (Fearon *et al*, 2011).

A limitation of this work is the lack of a placebo-controlled design. Nonetheless, our results are interesting and indicate a finding consistent across the study that has not been previously described with other biologic or chemotherapeutic agents in various cancers, including cholangiocarcinoma where cachexia is one of the major causes of morbidity and mortality. Our results add to the evidence suggesting that selumetinib is a particularly promising compound in patients with biliary cancer, as previously published (Bekaii-Saab *et al*, 2011).

These potential benefits for muscle function or other outcomes of selumetinib and potentially of other MEK inhibitors remain to be tested in randomised trials. Future randomised trials with this group of agents should include prospective assessment of inflammatory markers such as IL-6 and other cytokines implicated in cachexia, as well as outcomes that may reveal benefits of skeletal muscle gain. It would be of interest to continue evaluating new targeted cancer therapies for potential actions on muscle. A potential survival benefit of cachexia therapy was raised by the study of Zhou *et al* (2010), who showed that blocking muscle wasting by antagonism of the action of myostatin can have significant beneficial effects on survival in an animal model of cachexia. This result is currently being tested in a randomised phase II trial in pancreatic cancer (Eli Lilly and Company, 2012).

## Figures and Tables

**Table 1:** Patient characteristics and muscle and adipose tissue response to cholangiocarcinoma therapy

	**Phase II study cholangiocarcinoma selumetinib therapygroup**	**Cholangiocarcinoma standard therapy group**
Patients, *n*	20	30
Gender, male, %	30	56.7
Stage	100% Stage IV	100% Stage IV
Scan interval, days median (s.e.)	91.5 (6.7)	85.5 (51.1)
Time to death, days median (s.e.)	295 (4.5)a	277 (56.1)a
Age, mean±s.d.	54.5±14.4	58.6±12.2
Body mass index, kg m^−2^, mean±s.d.^#^	31.2±9.4	25.9±5.0
*Muscle change/100 days cm* ^*2*^		
Mean (s.d.)	13.80 (11.9)a	−7.3 (14.3)b
Estimated, kg	2.3	−1.2
*Adipose tissue change/100 days cm* ^*2*^		
Mean (s.d.)	−97.2 (413.2)a	−56.2 (85.4)a
Estimated kg	−6.6	−3.8

a, b comparison of tumour groups, means followed by different alphabets are different (*P*<0.05). Estimated kilograms of muscle and adipose are calculated from the regression equations reported by Shen *et al* (2004).

# Body mass index available for *N*=15 cholangiocarcinoma patients.
